# Leveraging a smartphone to perform time-gated luminescence measurements

**DOI:** 10.1371/journal.pone.0293740

**Published:** 2023-10-30

**Authors:** Brian E. Fratto, Emma L. Culver, Gabriel Davis, Robert Deans, John B. Goods, Sean Hwang, Nicole K. Keller, John A. Lawrence, Alexander R. Petty, Timothy M. Swager, Joseph J. Walish, Zhengguo Zhu, Jason R. Cox

**Affiliations:** 1 C2Sense, Inc., Watertown, Massachusetts, United States of America; 2 Department of Chemistry, Massachusetts Institute of Technology, Cambridge, Massachusetts, United States of America; Oregon State University, UNITED STATES

## Abstract

Empowered by advanced on-board sensors, high-performance optics packages and ever-increasing computational power, smartphones have democratized data generation, collection, and analysis. Building on this capacity, many platforms have been developed to enable its use as an optical sensing platform for colorimetric and fluorescence measurements. In this paper, we report the ability to enable a smartphone to perform laboratory quality time-resolved analysis of luminescent samples via the exploitation of the rolling shutter mechanism of the native CMOS imager. We achieve this by leveraging the smartphone’s standard image capture applications, commercially available image analysis software, and housing the device within a UV-LED containing case. These low-cost modifications enable us to demonstrate the smartphone’s analytical potential by performing tasks ranging from authentication and encryption to the interrogation of packaging, compounds, and physical phenomena. This approach underscores the power of repurposing existing technologies to extend the reach and inclusivity of scientific exploration, opening new avenues for data collection and analysis.

## Introduction

The ubiquity of smartphones, combined with the fusion of advanced camera, computational and sensing hardware, has changed the lives of billions worldwide [1–11]. The ability to communicate freely, create and stream live videos, and synchronously store and retrieve data are now widespread thanks to these advances [1, 3, 4]. The applicability of smartphones for portable scientific use cases is only recently emerging as an area of active commercial interest [2–6, 8–10] despite the obvious advantages of combining portable communication capabilities with sensor fusion. For this vision to come to fruition, the smartphone’s pervasive convenience must merge with the robust and precise measurement functionalities found in laboratory-grade instrumentation [5, 12].

Fluorescence spectroscopy is a marquis laboratory analytical technique for the trace detection of chemical and biological substances [13]. Predicated on molecular excitation and the subsequent measurement of luminescence, fluorescence-based techniques exhibit remarkable selectivity and sensitivity. These properties have enabled its use in many diverse fields, including biochemistry, molecular biology, environmental monitoring, and diagnostic platforms [14–17]. Yet, its complex and costly nature often restricts fluorescence spectroscopy largely to laboratory environments—the availability of portable, accessible variants remains elusive [18–21]. Traditional fluorescence measurements often rely on the quantification of emission intensity signals [22]. This is easily accomplished in laboratory settings where the optical geometry and lighting conditions are well controlled and sample preparation is easily accomplished prior to measurement. In the field, however, these measurements become markedly more difficult due to the non-standardized nature of interrogation [23, 24]. Reliable quantification of emission intensity using a smartphone would require the adoption of unchanging and well-calibrated ratiometric signals to mitigate the impact of the inverse square dependence of optical intensity on distance [23] Compounding the measurement further is the existence of multiple sources of autofluorescence that are ubiquitous in the field.

Time-gated spectroscopy, however, offers a promising alternative [25–35]. By measuring the emission lifetime of a luminescent material, this technique retains many of the benefits associated with fluorescence spectroscopy, namely exquisite sensitivity and selectivity, while proving less susceptible to radiative interference and eliminating the need for ratiometric signals [36, 37]. Since the luminescent substance’s lifetime is unique and typically invariant to read angle and/or ambient lighting conditions it offers resilience. Most importantly, measurements of delayed emission provide a means to overcome the negative effects of autofluorescence from the analyte’s local environment, one of the most significant challenges in realizing luminescence measurements outside of the laboratory [38]. By waiting for the relatively fast emissions of surrounding materials to decay before analysis, the signal to noise ratio of the measurement is significantly improved. Smartphone-based luminescent readers have been reported [39–52], their inability to conduct time-resolved measurements leaves them prone to the parent technique’s limitations. Consequently, we set our sights on enabling common smartphones—via low-cost enhancements to their native hardware and software—to perform time-delayed measurements, thus capitalizing on these ubiquitous devices’ inherent features in a cost-effective, user-friendly manner.

The current generation of smartphones, armed with a Complementary Metal-Oxide Semiconductor (CMOS) imaging chip, offer exceptional imaging capabilities at a consumer-friendly price point [53]. Despite their mass deployment, CMOS-based cameras have their limitations, chiefly their lack of a memory buffer, which confines the exposure process to an electronic ‘rolling shutter’ mechanism [43, 54, 55]. This mechanism operates through the sequential exposure and readout of each photodiode array row, resulting in a delay from the first to the last pixel row. Put another way, the rolling shutter mechanism means each row of the photodiode array is sampling a different time domain than the adjacent rows before and after. This lag often manifests as banding and geometric distortion, particularly when capturing fast-moving objects or intermittently flashing LEDs [54, 56, 57],—generally considered a photographic disadvantage [58]. Recently, we [59, 60] and others [52] have shown that these limitations can be exploited as features to perform time-delayed fluorescence measurements. In this paper, we extend the applicability of this technique to sensing and authentication via the development of a fully integrated smartphone device.

## Materials and methods

### Chemicals

The luminescent materials used in this manuscript are: (1,10-phenanthroline)tris[4,4,4-trifluoro-1-(2-thienyl)-1,3-butanedionato]europium(III) (Synonyms: Eu(tta)_3_phen), terbium 2,2,6,6-tetramethyl-3,5-heptanedionate, poly(2,5-di(3’,7’-dimethyloctyl)phenylene-1,4-ethynylene), N,N’-bis(1-hexylheptyl)perylene-3,4,9,10-tetracarboxybisimide, poly(9,9-dioctylfluorene-alt-benzothiadiazole) (CAS: 210347-52-7), and 4,4′-bis(9-carbazolyl)benzophenone. Where applicable, polymer matrices were selected based on their properties [61–64]. The matrix materials were poly(methyl methacrylate), Mw ~120,000 by GPC, and polystyrene, Mw 35,000. Toluene was used for all experiments. 4,4′-bis(9-carbazolyl)benzophenone is commercially available from Hoffman Fine Chemicals (Victoria, Australia), while all other chemicals are commercially available from Sigma-Aldrich (St. Louis, MO, USA). Additional Details are included in the Supporting Information, S1 File.

#### Optical measurements

Interrogation of the luminescent samples was conducted using our smartphone-based prototype device incorporating a custom printed circuit board able to pulse the UV LED contained on the board in response to a triggering event (the smartphone’s camera flash) (see Fig 2A). In earlier experiments, a Koolertron DSS Signal Generator Counter (60MHz High Precision Dual-channel Arbitrary Waveform Generator) was used to set the strobe frequency, waveform, and duty cycle of the excitation source. The signal generator was interfaced with a Zk-SJVA-4X 35W Step Up/Down Constant Current Converter Power Supply, and a XY-GMOS 10A MOSFET Motor driver and HIGH LOW trigger switch module, to control and power the excitation source. A Vishay 365nm LED (VLMU3510-365-130) was selected as the excitation source for this work. The effect of the LED’s rise and decay time was mitigated by the selection of a square waveform with a 50% duty cycle. Images were captured with the 26mm camera on iPhone model(s) 11 and 12 that were purchased from Apple Inc. (Cupertino, CA, USA). Additional Details are included in the Supporting Information, S1 File.

#### Image analysis

ImageJ (Version: 2.1.0/1.53c, build 5f23140693) and the smartphone compatible, browser based ImageJ.JS (https://ij.imjoy.io) were used for the analysis of captured images. Analysis involved separating the captured RGB image into individual color channels. Once separated into individual color channels; the plot profile function was used to interrogate the grayscale image(s). Additional Details are included in the Supporting Information, S1 File.

#### Luminescent samples

Solution-based samples (Fig 2B and 2C) comprised 0.1mM (1,10-phenanthroline)tris[4,4,4-trifluoro-1-(2-thienyl)-1,3-butanedionato]europium(III) and 0.0667mM N,N’-bis(1-hexylheptyl)perylene-3,4,9,10-tetracarboxybisimide dissolved in toluene.

**Fig 1:**
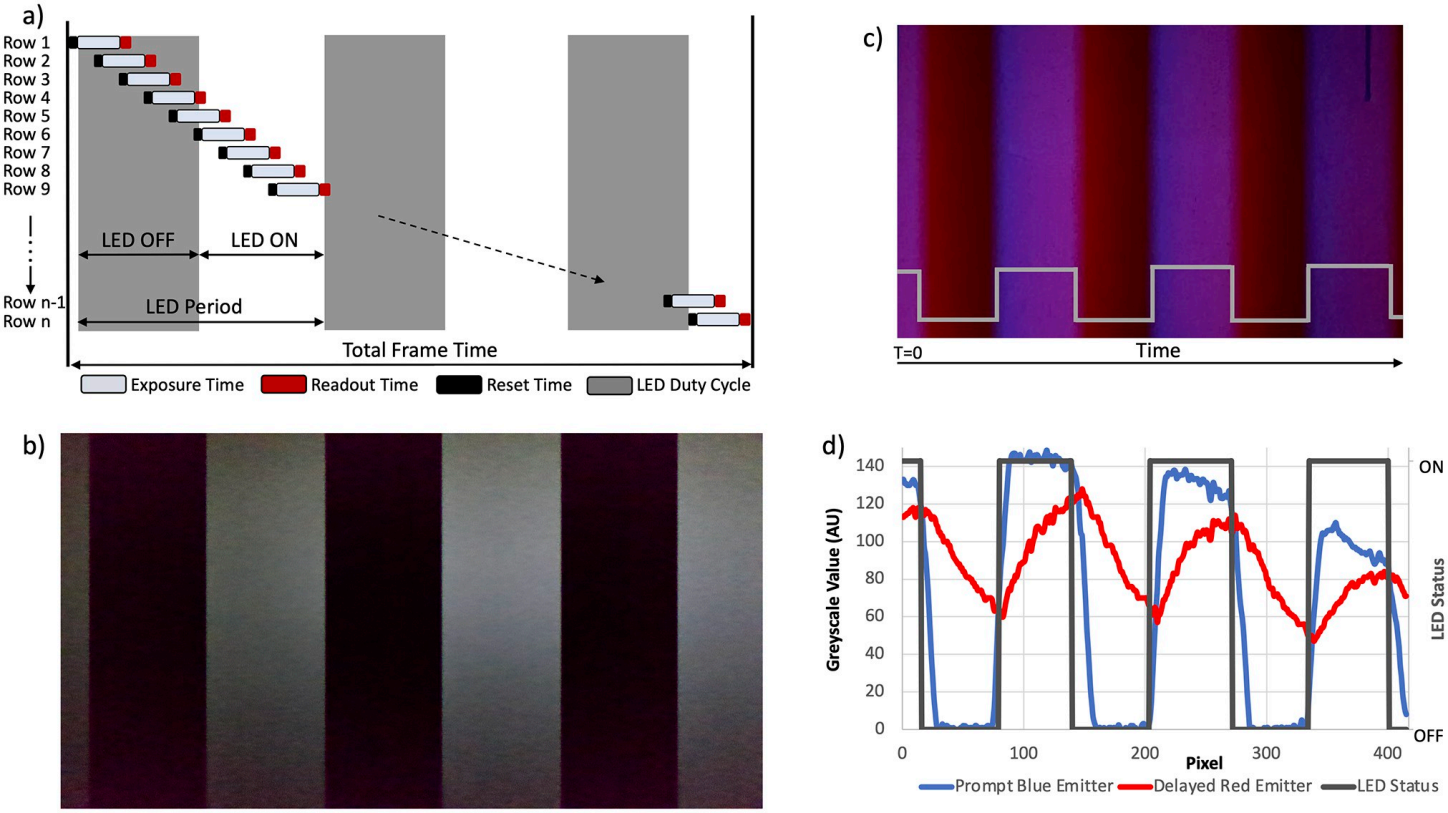
Visualization of the CMOS rolling shutter’s potential to measure time-gated signals. (A) Schematic representation of Rolling Shutter operation depicting the Exposure Time, Readout Time, and Reset Time. The duty cycle of a PWM modulated LED is also depicted (gray overlay). As illustrated, the rapid pulsing of the LED will only partially expose rows during the capture, resulting in a banded image. (B) Rolling Shutter based image of a pulsed LED captured using the 26mm camera of an iPhone 12. ISO 3200, Exposure 1/71429 s, *f*1.6. (C) A fast emitter, Poly(2,5-di(3’,7’-dimethyloctyl)phenylene-1,4-ethynylene), and slow emitter, Eu(tta)_3_phen, co-deposited on a solid support excited with a 365nm UV LED pulsed at 600.08 Hz. The image was captured using the 26mm camera of an iPhone 12. ISO 3,200, Exposure 1/71429 s, *f*1.6. (D) Analysis of On/Off transitions from the image shown in Fig 1C illustrating the presence of a fast emitter (blue) in the “on” band and slow emitter (red) in the “off” band.

The solid state sample, noted in Fig 1C, consisted of 1.0mM (1,10-phenanthroline)tris[4,4,4-trifluoro-1-(2-thienyl)-1,3-butanedionato]europium(III) and 0.25mg/ml poly(2,5-di(3’,7’-dimethyloctyl)phenylene-1,4-ethynylene) in a 0.166mM solution of poly(methyl methacrylate), dissolved in toluene and filtered (Whatman Puradisc 25 (Cat. No. 6747–2502, Lot. No. 17236201)). A Badger 105 Patriot airbrush (Franklin Park, IL, USA) was used to deposit approximately 3.72x10^-4^mL of solution per square centimeter. Dry nitrogen at a line pressure of 0.35 bar was used as the propellent with the airbrush held 15cm from the substrate.

The item illustrated in Fig 3, is composed of three unique toluene-based formulations. The red emissive material’s formulation contained 1.0mM (1,10-phenanthroline)tris[4,4,4-trifluoro-1-(2-thienyl)-1,3-butanedionato]europium(III) and 0.166mM poly(methyl methacrylate). The green emissive material’s formulation contained 35.0mM terbium 2,2,6,6-tetramethyl-3,5-heptanedionate contained 0.166mM poly(methyl methacrylate). The yellow/green emissive material’s formulation contained 0.025mg/mL poly(9,9-dioctylfluorene-alt-benzothiadiazole) and 0.166mM poly(methyl methacrylate). After dissolving, the solution(s) were filtered (Whatman Puradisc 25 (Cat. No. 6747–2502, Lot. No. 17236201)). The materials were individually deposited onto cardstock via a Techcon TSR2401 Automated Gantry Robot with a TS5540-MS spray-coating valve. The Techcon was run at a rate of 100mm/second with a 50mm Z-axis offset. The machine’s pneumatic activation pressure was set to 5.5 bar with a feed pressure of 1.03 bar. Additional Details are included in the Supporting Information, S1 File.

#### Oxygen / Pressure sensing

The sensing materials, as noted in Fig 4A, consisted of 1.90mM of 4,4′-bis(9-carbazolyl)benzophenone in a 1.43mM solution of polystyrene, dissolved into toluene. This formulation was deposited with a Badger 105 Patriot airbrush (Franklin Park, IL, USA) at a rate of 0.013mL/cm^2^ onto Avery Shipping Address Labels (manufacturers part no. 5135). Dry nitrogen with a line pressure of 0.25 bar was selected as the propellent, with an offset of 15cm between the airbrush and the selected substrate. Samples were dried under high vacuum for a minimum of 2h.

The testing setup consisted of an Edwards RV5 rotary vane vacuum pump, standard Swagelok fittings, and Chemglass glassware. An Omega battery powered digital pressure gauge (Measuring Range: -1.0 to 345.0 Millibar, Accuracy: ±0.5% FS, Stability: ±0.1% FS per year) was used to monitor and calibrate the system. The use of vacuum as a proxy for oxygen concentration has been previously demonstrated [65–68]. Additional Details are included in the Supporting Information, S1 File.

#### Temperature sensing

The temperature sensors were created by spin-coating onto DYMO Permanent Polyester Labels (Part number 1734523), using a KW-4A Spin Coater from Setcas LLC (San Diego, CA, USA), 1.0mM (1,10-phenanthroline)tris[4,4,4-trifluoro-1-(2-thienyl)-1,3-butanedionato]europium(III) dissolved in a 0.166mM solution of poly(methyl methacrylate) in toluene. After filtration (Whatman Puradisc 25 (Cat. No. 6747–2502, Lot. No. 17236201)), 70μl of solution was deposited onto a 24mm x 24mm label and spun at 3000rpm for 45 seconds.

The sensors (ten replicates) were affixed to a 3105-H24 aluminum alloy (thickness = 0.6858mm) plate and suspended inside a thermal chamber (TPS, Model No. TUJR-A-F4T, Temperature Range: -68°C to +180°C). Measurements were collected from approximately 15cm away under ambient and dark lighting conditions using the prototype device. The experimental conditions (-25°C to +100°C) were confirmed using a thermal imaging infrared camera (Teledyne FLIR, Model: FLIR E8). Additional Details are included in the Supporting Information, S1 File.

## Results and discussion

To unlock the smartphone camera’s potential to measure time-gated signals, we started by examining the CMOS rolling shutter’s reproducibility and fidelity using a 365nm LED pulsed at 600.08 Hz during image capture. The resulting photograph, as shown in Fig 1B, exhibited the characteristic banding artifact—a visualization of the LED’s “on-off” cycle—produced by a rolling shutter sensor observing a pulsed LED reflecting off a surface. The symmetry of the band widths corresponds to the LED duty cycle (preset here to 50%), with the total number of “on-off” cycles directly tied to the pulse frequency.

Fig 1C shows a non-fluorescent substrate coated with two emissive dyes: a fast emitter with an emissive lifetime of less than a nanosecond, and a slow emitter with a microsecond-range emissive lifetime. Upon strobing with a 365nm LED, the resulting “on” band contains emission from both the fast (blue) and slow (red) emitters, while the “off” band only contains the slow (red) emitter’s emission. Pixel-based color analysis of the captured image validates these observations.

Using commercially available image analysis software, we can extract a wealth of data, as illustrated in Fig 1D the graph illustrates the “on” and “off” bands. When the pulsed 365nm LED is on, the fast-emitting dye is excited and emits blue light, but when the LED is off, the dye quickly relaxes; essentially following the LED’s duty cycle. In contrast, the slow emitter, due to its longer lifetime, does not mimic the LED’s duty cycle. Overlaying the different color channels with the LED’s status highlights this (Fig 1D). Curve-fitting the intensity profile of the slow-emitter yields its decay constant, aligning well with the reported literature value. To the best of our knowledge, this marks the first instance of time-delayed measurements executed via a smartphone.

This experiment also allowed us to identify which luminescent materials were a good fit for the system, given that the CMOS imager’s time resolution becomes the limiting factor when detecting fast emitters. Consequently, this work made use of phosphorescent and time-delayed fluorescent dyes, which display emissions in the micro- to millisecond range. Specifically, we focused on commercially available lanthanides [69, 70], thermally activated delayed fluorescent (TADF) organic materials [71, 72], and fluorescent dyes.

**Fig 2:**
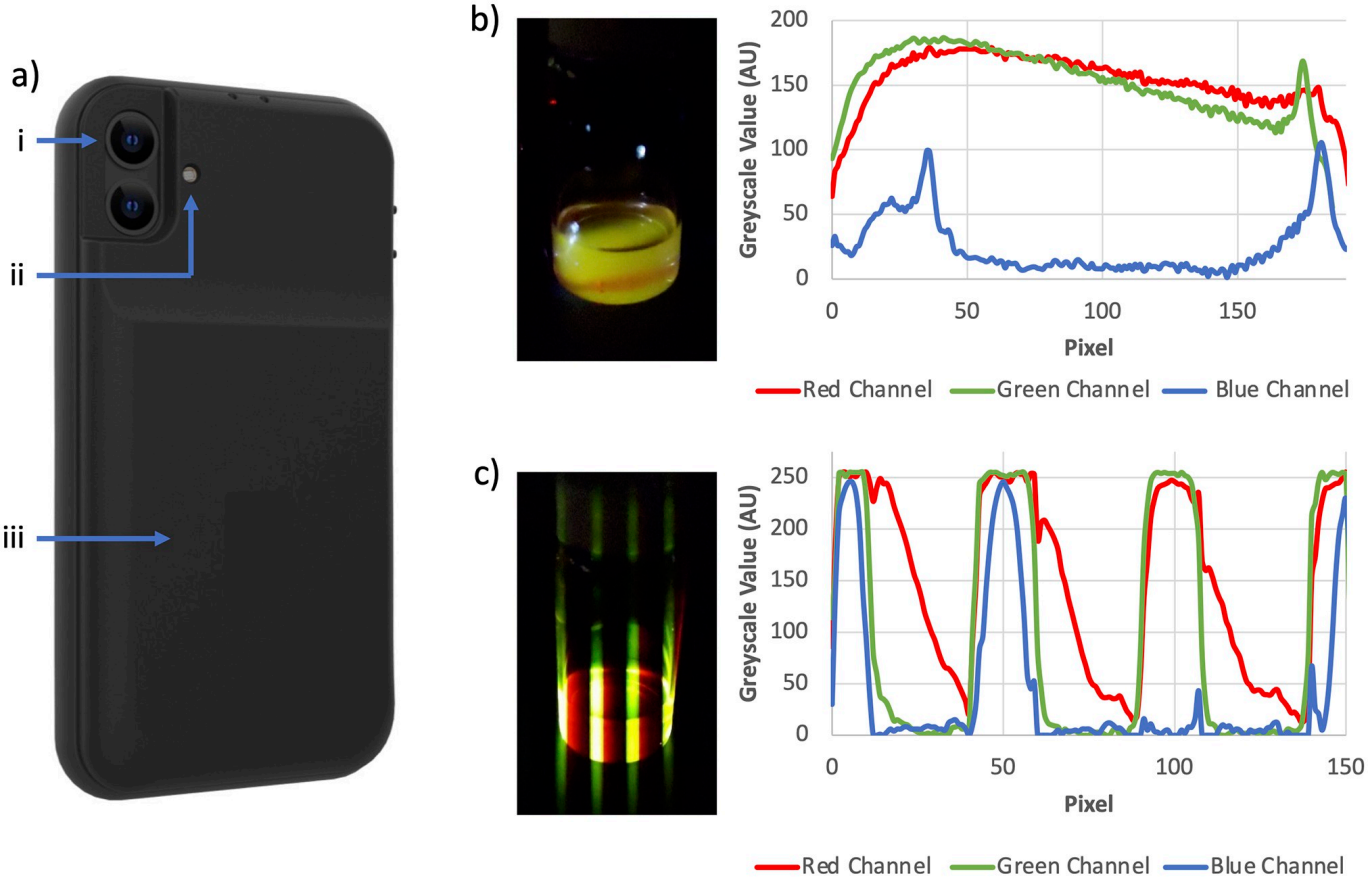
The integrated smartphone-based prototype and its function as a low-cost reader. (A) The integrated prototype illustrated with callouts for: i) the smartphone camera, ii) the LED excitation source, and iii) the case including rechargeable battery to power the LED. (B) A homogeneous mixture of fast emitter, N,N’-Bis(1-hexylheptyl)perylene-3,4,9,10-tetracarboxybisimide and slow emitter, Eu(tta)_3_phen dissolved in toluene. The image and subsequent analysis illustrate the system’s inability to differentiate between the fast and slow emitter when excited by constant 365nm illumination. (C) The same solution (as shown in 2b) interrogated with pulsed 365nm illumination. Both the image and analysis illustrate the system’s ability to deconvolute the fast emission (green/yellow) from the slow emission (red).

We then developed an integrated, field-deployable prototype modeled on a typical smartphone case (Fig 2A). This device allows the smartphone to work in concert with strobed LED excitation sources. The case makes use of the original equipment manufacturer (OEM) smartphone cameras (i), includes a generic solder pad for the LED excitation source (ii), houses the LED’s driver-board and power source (iii), and allows the device to function as a self-contained handheld unit.

The case design was optimized for the 26 mm camera with the LED’s position adjusted to align with the camera’s field of view. This setup enabled both luminescence and colorimetric images to be collected and analyzed via the readily available ImageJ software.

We then performed experiments to test the smartphone-based device’s ability to function as a low-cost reader. The results of these tests are shown in Fig 2B and 2C, comparing steady-state (constant illumination) and rolling shutter (pulsed illumination) images of non-aqueous solutions. The solution contained two different commercially available dyes with distinct emissive wavelengths and lifetimes, allowing us to compare steady-state fluorescence and lifetime-based measurements. The steady-state image illustrates the combined emission resulting from 365nm excitation, thereby preventing species differentiation in this complex mixture (Fig 2B). As a result, it is difficult to perform quantitative measurements or extract the decay constants.

In contrast, our device can differentiate the two species based on their lifetimes (Fig 2C). As illustrated, the emission from the fast emitter, N,N’-Bis(1-hexylheptyl)perylene-3,4,9,10-tetracarboxybisimide, directly correlates with the LED’s duty cycle. This confines the fast emission to the "on" band. Meanwhile, the slow emission from Eu(tta)_3_phen does not correlate with the device’s LED duty cycle and appears in the "off" band. Analysis of the emission allows for decay constant calculation and positive identification of the species.

Further vetting of our device’s function as a low-cost reader made use of lanthanide-based complexes. Lanthanide complexes are often used as security inks for products, documents, and currency due to their resistance to photobleaching, narrow emission profiles, and reduced self-quenching [73–75]. The inks allow for the insertion of hidden information into spatial patterns via their emission wavelengths and luminescent lifetimes. Current methods for analyzing these inks include simple visual inspection (presence/absence) or more complex laboratory-based analysis. Existing approaches fail to fully exploit the potential of these materials and/or increase the cost of analysis, thereby limiting their commercial impact.

**Fig 3:**
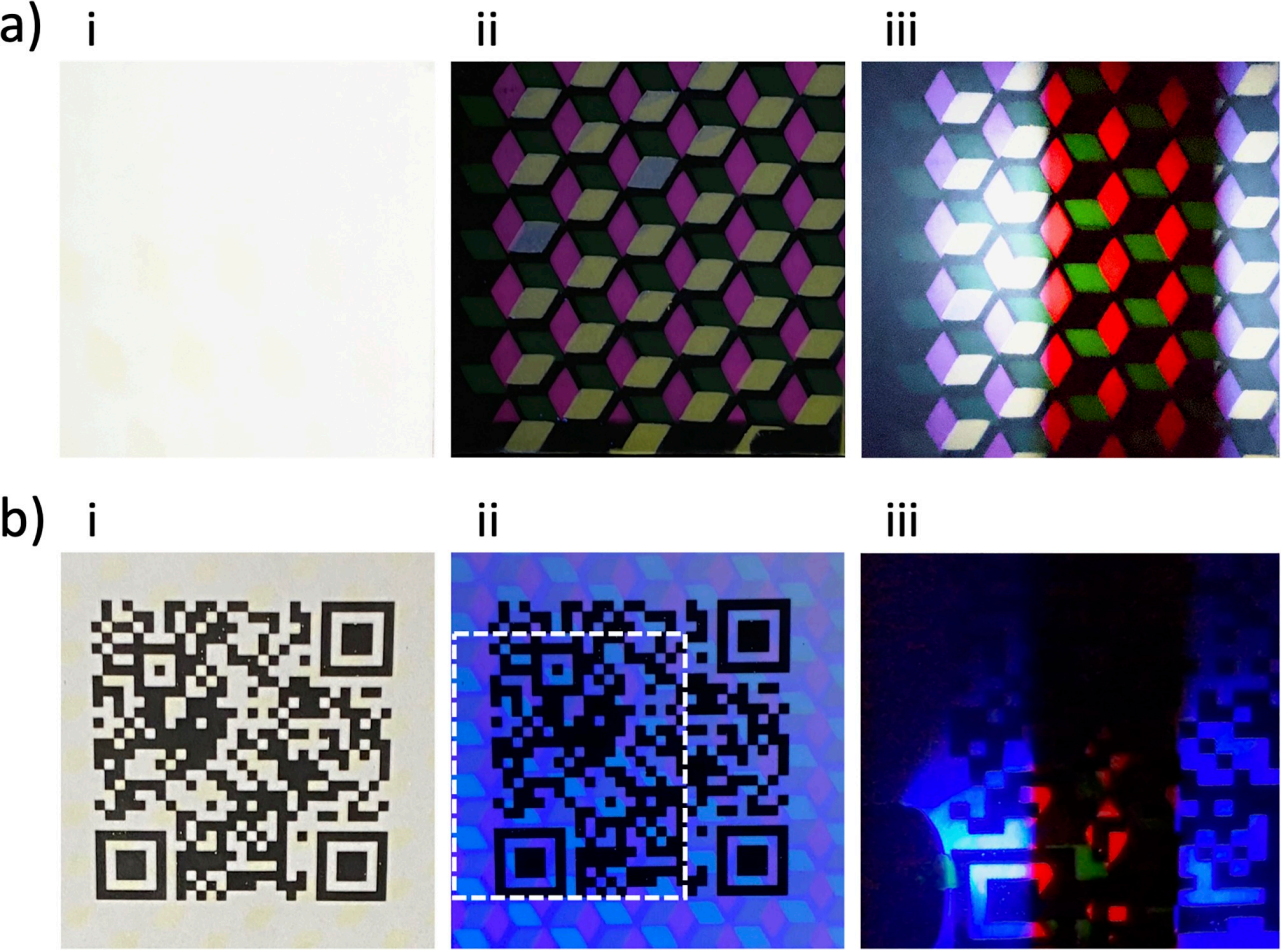
Luminescence and lifetime analysis of lanthanide security inks via the smartphone-based device. (A) Geometric patterning of a fast emitter, Poly(9,9-dioctylfluorene-alt-benzothiadiazole) (yellow/green) and multiple slow emitters, Eu(tta)_3_phen (red) and Terbium 2,2,6,6-tetramethyl-3,5-heptanedionate (green). Interrogation under (i) ambient light, (ii) constant 365nm illumination, and (iii) strobed 365nm excitation with rolling shutter. (B) The same geometric pattern illustrated in Fig 3A combined with a QR code to enable multiple authentication techniques. Under (i) ambient light only the QR code is visible. Constant illumination (ii) by 365nm reveals both the QR code and the pattern’s fast emission. Figure (iii) demonstrates strobed 365nm excitation and imaging with rolling shutter.

Our smartphone-based device addresses these issues by enabling both luminescence and lifetime analysis of lanthanide security inks. Its versatility was demonstrated by fabricating tags that were invisible to the naked eye, function as typical security tags under steady-state UV illumination and reveal hidden time-resolved patterns under pulsed UV illumination when analyzed by our device (Fig 3). This level of analysis was previously only possible with laboratory time-resolved instruments.

In addition to the analysis of lanthanide complexes, we vetted of our device’s ability to analyze TADF and phosphorescent materials for sensing applications. A well-known phenomenon in photochemistry is the quenching of long-lived excited states by molecular oxygen, enabling the quantitative measurement of oxygen concentrations based on the material’s emission [47, 76–80]. However, leveraging such materials as oxygen sensors has been problematic due to the hardware required to analyze the emitting species. Our smartphone-based prototype offers a portable and field-deployable solution for reading oxygen-sensitive delayed emitters.

**Fig 4:**
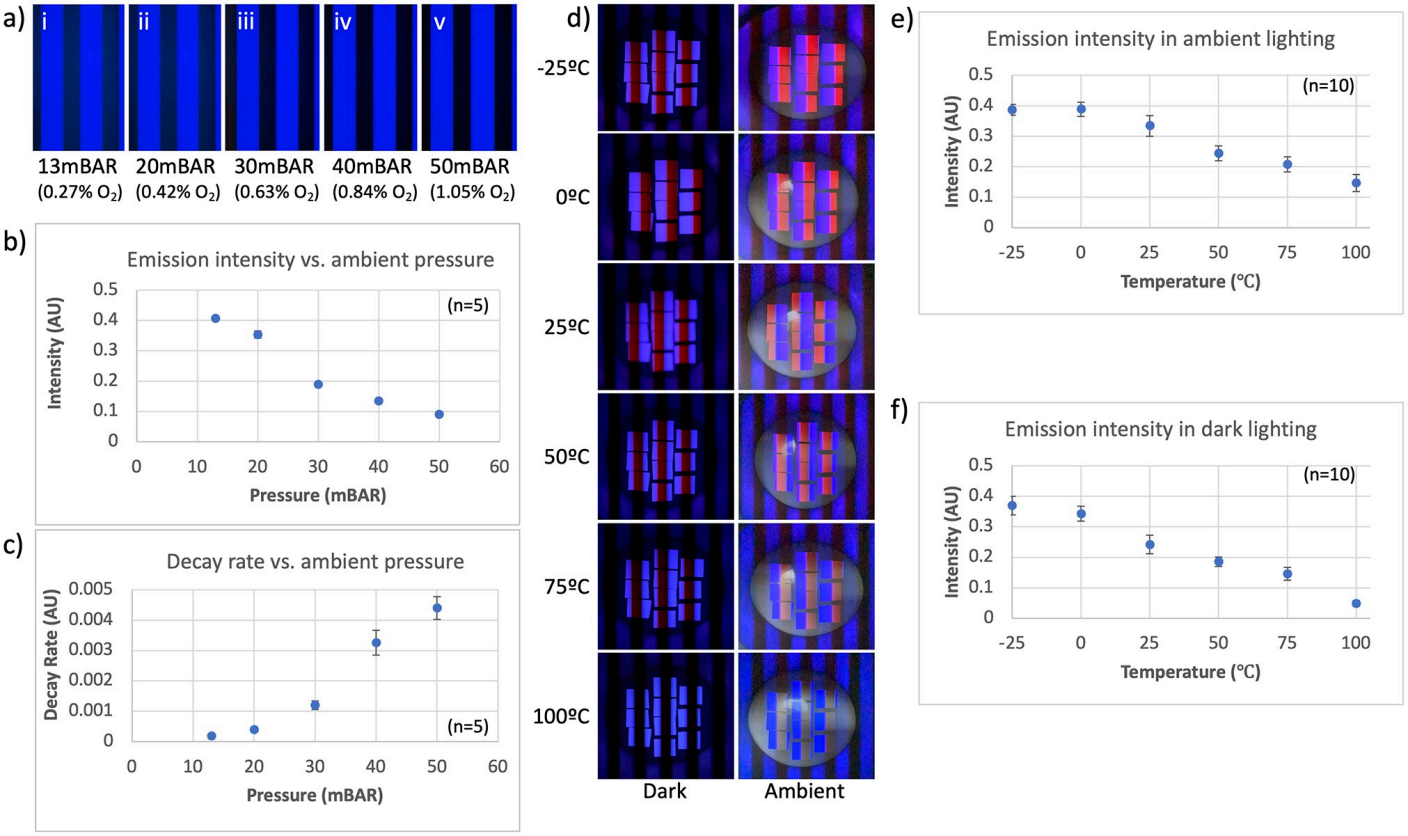
Analysis of oxygen concentration and temperature response via the smartphone-based device. (A) A Tyvek support coated with 4,4′-bis(9-carbazolyl)benzophenone in polystyrene. Sensing is illustrated in i–v where the tag has been exposed to different amounts of oxygen. (B) Emission intensity as a function of ambient pressure. (C) Decay rate as a function of ambient pressure. (D) Temperature response of Eu(tta)_3_phen in PMMA imaged under ambient and dark lighting conditions. (E) Emission intensity as a function of temperature imaged under ambient lighting. (F) Emission intensity as a function of temperature imaged under dark lighting.

As demonstrated in Fig 4A, a well-known TADF material, 4,4′-bis(9-carbazolyl)benzophenone, was embedded in an oxygen-permeable matrix [57, 58], deposited on a substrate and exposed to various ambient oxygen concentrations by reducing the ambient pressure [65–68]. As shown in panels i–v, the emission from this material was variably quenched based on the amount of oxygen present. Using our device, we could capture images obtained under different pressures/oxygen concentrations. Analysis of these images enables the calculation of the resultant intensity and decay rates, enabling non-invasive, line-of-sight oxygen sensing (Fig 4B and 4C). Additional testing vetted the device’s ability to function under different lighting conditions. As depicted in Fig 4D, we monitored the response of a temperature responsive material (exhibiting delayed emission) under ambient and dark conditions. In both cases the resulting lifetime data correlated with the ambient temperature (Fig 4E and 4F), further highlighting the smartphone-based prototype’s function as a portable and field-deployable solution.

## Conclusion

In conclusion, we have demonstrated that conventional smartphones can be leveraged to perform exquisite time-delayed measurements. Coupling the smartphone’s rolling shutter mechanism with materials that exhibit long-lived emissions and pulsed excitation sources, enables the differentiation of materials based on their emissive lifetimes.

We present here several examples, including images of pulsed LEDs and emitters with different luminescent properties, illustrating the versatility and sensitivity of the system. Importantly, we also demonstrate that the system can perform quantitative measurements, thereby yielding valuable information about the concentration of analytes present.

The simplicity, portability, and affordability of this approach make it a promising tool for many application areas and opens new possibilities within the field of imaging for both academia and industry. We hope that our work encourages further exploration of smartphone-based luminescence imaging, accelerates its adoption, and inspires innovation via increased access to analytical techniques previously restricted to laboratory settings.

## Supporting information

S1 File Its contains additional information about the chemicals, deposition and interrogation methods used. (DOCX)
