# Supplementary material for: Leveraging a smartphone to perform time-gated luminescence measurements
Source: PLoS One. 2023 Oct 30;18(10):e0293740. doi: 10.1371/journal.pone.0293740 (PMC10615318; doi:10.1371/journal.pone.0293740)
Supplement: S1 File — (DOCX) [file pone.0293740.s001.docx]

Leveraging a Smartphone to Perform Time-gated Luminescence Measurements

**Supplementary Material**

**Materials and Methods**

## With an overall aim of demonstrating the capability of the Rolling Shutter for the interrogation of targets, this work primarily utilized commercially available, commodity chemicals. When creating these targets, the main luminescent materials used were (1,10-Phenanthroline)tris[4,4,4-trifluoro-1-(2-thienyl)-1,3-butanedionato]europium(III) (Synonyms: Eu(tta)_3_phen, CAS: 17904-86-8), terbium 2,2,6,6-tetramethyl-3,5-heptanedionate (CAS: 15492-51-0), poly(2,5-di(3’,7’-dimethyloctyl)phenylene-1,4-ethynylene) (CAS: 211809-71-1), N,N’-Bis(1-hexylheptyl)perylene-3,4,9,10-tetracarboxybisimide (CAS: 110590-84-6), Poly(9,9-dioctylfluorene-alt-benzothiadiazole) (CAS: 210347-52-7), and 4,4′-bis(9-carbazolyl)benzophenone (CAS: 1030630-68-2). The solvent used for all work was toluene (CAS: 108-88-3).

In practice, when the luminescent materials were not being interrogated in solution, they were incorporated into matrices that were selected for their specific properties. [S1, S2] The matrix materials used were poly(methyl methacrylate), Mw ~120,000 by GPC (CAS: 9011-14-7) and polystyrene, Mw 35,000 (CAS: 9003-53-6) which were deposited on various host substrates. In line with the use of commercially available materials, commercially scalable deposition and coating methods were prioritized such as spray coating and spin coating.

## Interrogation with the Rolling Shutter was enabled via the use of a prototype (Figure 2A) and/or the pairing of an iPhone with a LED. Initial experimentation made use of a Koolertron DSS Signal Generator Counter (60MHz High Precision Dual-channel Arbitrary Waveform Generator), a Zk-SJVA-4X 35W Step up/Down Constant Current Converter Power Supply, and a XY-GMOS 10A MOSFET Motor driver and HIGH LOW trigger switch module. As implemented, the Koolertron DSS Signal Generator was used to set the strobe frequency, waveform, and duty cycle of the excitation source. Images illustrated in Figures 1 to 4 were all captured utilizing a 365nm LED as an excitation source. Rolling Shutter images were initially captured on an Apple iPhone 11 with the iOS 13 operating system. As testing progressed, a prototype device was created (Figure 2A). This device, based on the benchtop strobing test rig, was created to enable portable, handheld interrogation of targets. As designed, a minimalist case was fabricated to house an excitation source (LED), its requisite hardware and a battery. This Prototype was deployed as a handheld sensing platform, superseding the benchtop apparatus. Although various LEDs can be fitted into this device to yield different excitation wavelengths and/or power profiles, the experiments noted in Figures 1 to 4, utilize a case that has been configured to include 500mW, 365nm LED.

## Experimental Visualization of the Rolling Shutter Effect

## As described, the recovery of accumulated signal from a Complementary Metal-Oxide Semiconductor (CMOS) is tied to a measurement unit in each pixel. Although this recovery may occur at a single time, as is the case with a Global Shutter, an electronic rolling shutter will expose and read each row of the photodiode array individually. [S3] Without the presence of a memory buffer in a CMOS chip, this mechanism results in a time delay between the readout of the last pixel in the first row and the last pixel in the final row. [S4] Experimentally this may be visualized via the use of an LED that is strobed at a higher frequency than the CMOS’s Frame Rate (Figure 1B).

## To visualize crisp transitions between the “on” and “off” state, the DSS generator was setup to drive the 365nm LED with a duty cycle that is representative of a square wave form. The duty-cycle of the wave form was selected to be 50%. Selection of the strobing frequency was experimentally determined to be 450 h/z by sweeping the strobing frequency while monitoring the real-time image being generated by the iPhone. The resulting image, captured with an iPhone 12, (ISO: 3,200, Exposure time: 1/71429, *f*/1.6) is illustrated in Figure 1B.

## Creation of Luminescent Samples

## To create luminescent targets for interrogation with the Rolling Shutter, materials were surveyed to determine their excitation/emission profiles, possibility of delayed luminescence and commercial viability. The specific formulations which pertain to Figures 1 to 4 are noted in the subsequent sections.

## Solution based samples, as seen in Figure 2B and 2C, were created with the use of Eu(tta)_3_phen as the delayed emitter. The excitation and emission profile of this lanthanide coordination complex enabled the emission of the molecule to be confined to the red channel of the CMOS imager. By selecting N,N’-Bis(1-hexylheptyl)perylene-3,4,9,10-tetracarboxybisimide as a prompt emitter, the formulated solution is able to show both prompt and delayed emission. Specifically, 0.1mM Eu(tta)_3_phen and 0.0667mM N,N’-Bis(1-hexylheptyl)perylene-3,4,9,10-tetracarboxybisimide were dissolved in toluene via sonication. The sample, as illustrated in Figure 2B and 2C, was excited with both a steady state and a strobed 365nm LED. The resulting images were then analyzed with the “plot profile” function, a standard feature of ImageJ (Version: 2.1.0/1.53c, Build 5f23140693) and the smartphone compatible, browser based ImageJ.JS (https:/ij.imjoy.io). Analysis of the samples included the separation of the color channels and the plotting of the images profile to obtain a greyscale value (AU) of the image. Plotting of this analysis enables the deconvolution of the different emitters which emphasizes the delayed emission in the Rolling Shutter image vs the image of steady state illumination.

## Authentication

Solid state samples were also formulated to enable the visualization of the rolling shutter (Figure 1C), authentication of a sample, and sensing applications (Figures 3 and Figure 4). The polymer matrix was selected based on the final application of the sensor. Authentication based samples utilized Poly(methyl methacrylate), as a polymer matrix for its optical clarity. Eu(tta)_3_phen was selected as the delayed emitter for the tagged item illustrated in Figure 1C. In this application, a prompt-emitter was implemented to aid in the determination of the “On” band. Based on its emission profile, Poly(2,5-di(3’,7’-dimethyloctyl)phenylene-1,4-ethynylene) was used as the prompt-emitter. The final toluene-based formulation included 1.0mM Eu(tta)_3_phen and 0.25mg/ml Poly(2,5-di(3’,7’-dimethyloctyl)phenylene-1,4-ethynylene) in a 0.166mM solution of Poly(methyl methacrylate), in Toluene. Upon dissolution of all materials, the resulting formulation was filtered through a Whatman Puradisc 25 (Cat. No. 6747-2502, Lot. No. 17236201) to ensure homogeneity and removal of any non-dissolving solids. In the case of Figure 1C, the host substrate was spray coated with a Badger 105 Patriot airbrush with a deposition rate of 3.72x10^-4^ml/cm^2^, a line pressure of 0.35 bar was selected with the airbrush being kept a distance of 15cm from the host substrate. The pressure and distance were selected to ensure full atomization and control over droplet size. [S5] To minimize moisture-induced nucleation, dry nitrogen was selected as a propellent. Although not required, the samples were placed under high vacuum for a minimum of 1 hour to standardize drying.

Additionally, tags with multiple delayed-emission components were created. As illustrated in Figure 3, these tags are comprised of a prompt emissive material as well as two materials with delayed emission. In contrast to the tag illustrated in Figure 1C, a unique formulation was created for each emissive material. Initially, a main solution of 0.166mM Poly(methyl methacrylate), in toluene was created. This solution was utilized as the solvent for all subsequent solutions. The red, delayed-emitting solution was created by dissolving Eu(tta)_3_phen in the 0.166mM Poly(methyl methacrylate), solution to yield a final concentration of 1.0mM Eu(tta)_3_phen. The green, delayed-emitting solution was created by dissolving Terbium 2,2,6,6-tetramethyl-3,5-heptanedionate in the 0.166mM Poly(methyl methacrylate), solution to yield a final concentration of 35.0mM Terbium 2,2,6,6-tetramethyl-3,5-heptanedionate. A final, prompt-emitting solution was created using Poly(9,9-dioctylfluorene-alt-benzothiadiazole). For this solution, Poly(9,9-dioctylfluorene-alt-benzothiadiazole) was dissolved in the 0.166mM solution of Poly(methyl methacrylate), solution to yield a final concentration of 0.025mg/ml. Prior to spray coating, the solutions were filtered with a Whatman Puradisc 25 (Cat. No. 6747-2502, Lot. No. 17236201). The multi-component pattern was subsequently spray coated onto a cardstock support via a Techcon TSR2401 Automated Gantry Robot with a TS5540-MS spray-coating valve. The Techcon device was run at a rate of 100mm/second with a 50mm Z-axis offset. The machine’s pneumatics were configured to have an activation pressure of 5.5 bar and a feed pressure of 1.03 bar. Although not required, the samples were placed under high vacuum for a minimum of 1 hour to standardize drying.

## Analysis of the samples included imaging under ambient lighting (iPhone 12 Pro, ISO: 3200, Exposure Time: 1/2020, *f/*1.6) and then with both a steady state and a strobed 365nm LED (iPhone 12 Pro, ISO: 3200, Exposure Time: 1/17241, *f/*1.6). As illustrated in Figure 3, the samples are invisible under ambient lighting, yet possess different patterns when illuminated with either a steady state or strobed excitation source.

## Sensing

## The formulation for sensing tags, noted in Figure 4, accounted for sensitivity and cyclicality. Although possible to monitor both dosimeters and single use sensors with the rolling shutter, the fidelity of the rolling shutter measurements pairs well with reversable sensors that enable a rapid, real-time response. For both Oxygen/Pressure sensing Figure 4A-C, and temperature sensing Figure 4D, the properties of the matrix and the presence (or lack thereof) of the support material was a main consideration.

## Oxygen/Pressure Sensing

The Oxygen sensor was created using 4,4′-bis(9-carbazolyl)benzophenone with a polystyrene (average Mw 35,000) matrix. The use of polystyrene enabled oxygen permeability (P_02_ = 0.0031 x 10^-13^ cm^3^ (stp) cm cm^-2^ s^-1^ PA^-1^) [S6] while also preventing the dyes from rotation, which has been reported to drastically increase TADF lifetimes and quantum yields. [S7] Specifically, the formulation consisted of 1.90mM of 4,4′-bis(9-carbazolyl)benzophenone which was dissolved into a 1.43mM of Polystyrene solution. Toluene was chosen as a solvent for the system based on its compatibility with multiple application methods. In the case of Figure 4A, Avery Shipping Address Labels (manufacturers part no. 5135) were selected as host substrate. These labels were spray coated with a Badger 105 Patriot airbrush at a deposition rate of 0.013ml/cm^2^. A line pressure of 0.25 bar was selected to ensure atomization and control droplet size. [S5] To minimize moisture-induced nucleation, dry nitrogen was selected as a propellent. The resulting samples were dried under high vacuum for a minimum of 2h.

The experimental rig was composed of an Edwards RV5 rotary vane vacuum pump with a brass integral bonnet needle valve (0.37 Cv, 1/4" in. Swagelok tube fitting, vee stem, Swagelok part number: B-1VS4) to tune the systems pressure. The system was monitored with a calibrated Omega battery powered digital pressure gauge (Measuring Range: -1.0 to 345.0 Millibar, Accuracy: ±0.5% FS, Stability: ±0.1% FS per year) as the meteorological ground truth. A Chemglass Drying Chamber (AF-0556-02) was used as the sample holder, enabling the prototype device to perform standoff measurements at 10cm in ambient lighting conditions. To ensure the cyclicality of the sensor, five replicates were conducted. Prior to each replicate, the sensor was exposed to ambient atmosphere for 180 seconds to ensure that the sensors were at equilibrium with the ambient atmosphere before the next replicate.

**Temperature Sensing**

The temperature sensor was created by spin-coating a toluene-based formulation onto DYMO Permanent Polyester Labels (Part number 1734523) which were cut to a size of 24mm x 24mm. Poly(methyl methacrylate), was selected as the matrix due to its optical properties across temperature ranges^1^. The Formulation for these sensors included 1.0mM Eu(tta)_3_phen and 0.166mM Poly(methyl methacrylate), in toluene. The subsequent solution was then filtered with a Whatman Puradisc 25 (Cat. No. 6747-2502, Lot. No. 17236201) to remove particulate. Spin coating was undertaken with a KW-4A Spin Coater from Setcas LLC. The machine was set to spin the samples at 3000rpm for 45 seconds. 70μl of the filtered formulation was deposited across the whole 24mm x 24mm surface area to ensured uniform coverage of the whole 24mm x 24mm sensor.

## To conduct the temperature measurements, 10 replicate sensors were affixed to a 0.6858mm thick piece of 3105-H24 aluminum alloy (Figure 4D). The support was fitted to the inside of a thermal chamber in an orientation that did not hinder standoff interrogation by the prototype device. The thermal chamber (TPS, Model No. TUJR-A-F4T, Temperature Range: -68°C to +180°C) enabled the samples to be exposed to a temperature range of -25°C to +100°C. Prior to each measurement, the temperature of the thermal chamber was verified via a thermal imaging infrared camera (Teledyne Flir, Model: FLIR E8) and adjusted as needed. Upon verification, the sensors were interrogated from 15.25cm in both dark and ambient lighting.

## Citations

## A Lalova A, Todorov R. Optical properties of thin PMMA films for sensor applications. Bulgarian Chemical Communications. 2015;47: 29-34.

1. Koren K, Hutter L, Enko B. Pein A, Borisov SM, Klimant I. Tuning the dynamic range and sensitivity of optical oxygen-sensors employing differently substituted polystyrene-derivatives. Sensors and Actuators B. 2013;176: 344-350.
2. Köhler S, Lovisotto G, Birnbach S, Baker R, Martinovic I. They see me rollin’: Inherent vulnerability of the rolling shutter in CMOS image Sensors. ACSAC ’21: Annual Computer Security Applications Conference (Association for Computer Machinery). https://doi.org/10.48550/arXiv.2101.10011.
3. Kuroda T, Essential Principles of Image Sensors, 1st ed. Boca Raton: CRC Press: 2015.

## Zabihi F, Eslamian M. Characteristics of thin films fabricated by spray coating on rough and permeable paper substrates. Journal of Coatings Technology and Research. 2015;12: 489-503

1. Brandrup J, Immergut EH, Grulke EA. Polymer Handbook, 2 Volumes Set. 4th ed, New York: Wiley; 2003.
2. A. Steinegger A, Klimant I, Borisov SM. Purely organic dyes with thermally activated delayed fluorescence – a versatile class of indicators for optical temperature sensing. Advanced Optical Materials. 2017;5: 1700372.
